# Contraction Type Influences Critical Ages for Declines In Lower Body Specific Force in Women Ages 20 to 89 Years

**DOI:** 10.1093/geroni/igaa057.602

**Published:** 2020-12-16

**Authors:** Ryan Miller, Eduardo Freitas, Aaron Heishman, Debra Bemben, Michael Bemben

**Affiliations:** University of Oklahoma, Norman, Oklahoma, United States

## Abstract

The ability for a muscle to produce force relative to its size, specific force, is an important characteristic for healthy aging. Few studies have identified the influence that different muscle groups or contraction types may have on the onset of declines in specific force. Therefore, the aims of the current study were to identify critical ages for changes in upper (quadriceps and hamstrings) and lower leg (soleus and tibialis anterior) specific force and to determine if the onset of decline is influenced by contraction type (isometric or dynamic) in women aged 20 to 89 years. One-hundred and fifty-two women (47.1±17.7years, 164.2±7.0cm, 67.1±10.7kg) matched for physical activity with approximately 10 participants per five-year interval (20-24, 25-29years, etc.), were included in the present analysis. Specific force was calculated from peak torque values measured from isometric (ISOM) or isokinetic testing (60deg/s and 240deg/s) and made relative to muscle area measured by peripheral quantitative computed tomography. An iterative segmental modelling approach was performed to identify critical age periods for changes in specific force. For the upper leg, ISOM and 60deg/s specific force was maintained across the lifespan, whereas 240deg/s revealed a critical age of 35.2±5.3years. Critical ages were identified for all contraction types for the lower leg and occurred earlier with increasing velocity (ISOM: 63±6.6years, 60deg/s: 53.9±4.5years, and 240deg/s: 49±3.9years). These data suggest that muscle groups of the leg do not display uniform changes across different contraction types and that high velocity contractions display earlier declines across the lifespan.

